# Transient gestational hypothyroxinemia accelerates and enhances ulcerative colitis-like disorder in the male offspring

**DOI:** 10.3389/fendo.2023.1269121

**Published:** 2024-01-04

**Authors:** Juan Carlos Rivera, Ma. Cecilia Opazo, Rosario Hernández-Armengol, Oscar Álvarez, María José Mendoza-León, Esteban Caamaño, Sebastian Gatica, Karen Bohmwald, Susan M. Bueno, Pablo A. González, Michel Neunlist, Helene Boudin, Alexis M. Kalergis, Claudia A. Riedel

**Affiliations:** ^1^ Laboratorio de Endocrino-inmunología, Departamento de Ciencias Biológicas, Facultad de Ciencias de la Vida, Universidad Andrés Bello, Santiago, Chile; ^2^ Millennium Institute on Immunology and Immunotherapy, Facultad de Ciencias Biológicas, Pontificia Universidad Católica de Chile, Santiago, Chile; ^3^ Facultad de Medicina Veterinaria y Agronomía, Instituto de Ciencias Naturales, Universidad de las Américas, Santiago, Chile; ^4^ Instituto de Ciencias Biomédicas, Facultad de Ciencias de la Salud, Universidad Autónoma de Chile, Santiago, Chile; ^5^ Facultad de Ciencias Biológicas, Pontificia Universidad Católica de Chile, Santiago, Chile; ^6^ Université de Nantes, Inserm, TENS, The Enteric Nervous System in Gut and Brain Disorders, IMAD, Nantes, France; ^7^ Departamento de Endocrinología, Facultad de Medicina, Pontificia Universidad Católica de Chile, Santiago, Chile

**Keywords:** gestational hypothyroxinemia, ulcerative colitis, colon inflammation, immune cells, autoimmunity, radical oxygen species

## Abstract

**Introduction:**

Gestational hypothyroxinemia (HTX) is a condition that occurs frequently at the beginning of pregnancy, and it correlates with cognitive impairment, autism, and attentional deficit in the offspring. Evidence in animal models suggests that gestational HTX can increase the susceptibility of the offspring to develop strong inflammation in immune-mediated inflammatory diseases. Ulcerative colitis (UC) is a frequent inflammatory bowel disease with unknown causes. Therefore, the intensity of ulcerative colitis-like disorder (UCLD) and the cellular and molecular factors involved in proinflammatory or anti-inflammatory responses were analyzed in the offspring gestated in HTX (HTX-offspring) and compared with the offspring gestated in euthyroidism (Control-offspring).

**Methods:**

Gestational HTX was induced by the administration of 2-mercapto-1-methylimidazole in drinking water to pregnant mice during E10–E14. The HTX-offspring were induced with UCLD by the acute administration of dextran sodium sulfate (DSS). The score of UCLD symptomatology was registered every day, and colon histopathology, immune cells, and molecular factors involved in the inflammatory or anti-inflammatory response were analyzed on day 6 of DSS treatment.

**Results:**

The HTX-offspring displayed earlier UCLD pathological symptoms compared with the Control-offspring. After 6 days of DSS treatment, the HTX-offspring almost doubled the score of the Control-offspring. The histopathological analyses of the colon samples showed signs of inflammation at the distal and medial colon for both the HTX-offspring and Control-offspring. However, significantly more inflammatory features were detected in the proximal colon of the HTX-offspring induced with UCLD compared with the Control-offspring induced with UCLD. Significantly reduced mRNA contents encoding for protective molecules like glutamate-cysteine ligase catalytic subunit (GCLC) and mucin-2 (MUC-2) were found in the colon of the HTX-offspring as compared with the Control-offspring. Higher percentages of Th17 lymphocytes were detected in the colon tissues of the HTX-offspring induced or not with UCLD as compared with the Control-offspring.

**Discussion:**

Gestational HTX accelerates the onset and increases the intensity of UCLD in the offspring. The low expression of MUC-2 and GCLC together with high levels of Th17 Lymphocytes in the colon tissue suggests that the HTX-offspring has molecular and cellular features that favor inflammation and tissue damage. These results are important evidence to be aware of the impact of gestational HTX as a risk factor for UCLD development in offspring.

## Introduction

The maternal thyroid hormones (THs) 3,5,3′-L-tri-iodothyronine (T_3_) and 3,5,3′5′-L-tetra-iodide-thyronine (T_4_) are essential for proper fetus development (1). Gestational hypothyroxinemia (HTX) clinically characterized by normal maternal T_3_ and thyroid-stimulating hormone (TSH) but low T_4_ serum levels has irreversible consequences over fetus development (2, 3). The prevalence of this condition fluctuates between 1.3% and 23.9% around the world (3, 4). The high frequency of gestational HTX is due to the maternal thyroid gland must increase TH production, especially during the first 20 weeks of pregnancy, to provide with THs to the mother and fetus (5). If maternal iodine intake is not enough for TH synthesis, the maternal thyroid gland can fail to produce THs, and the maternal biological system will compensate to keep normal T_3_ levels by decreasing T_4_ production and increasing T_3_ synthesis from T_4_ (6). Even though gestational HTX is harmless to the mother, several studies in humans have revealed that this condition can be detrimental to the offspring (7–15). It has been shown that the offspring gestated under HTX (HTX-offspring) increases the probability of developing attentional deficit disorder (16–18), low IQ (18), and autism (19). In addition, studies in mice have shown that the consequences of gestational HTX in the offspring surpass the central nervous system (CNS), increasing their susceptibility to have a strong inflammatory response upon induction of experimental autoimmune encephalomyelitis (EAE) (20). EAE is an inducible mouse autoimmune disease widely used as a model of multiple sclerosis (MS) (20). The molecular mechanisms that enhanced inflammatory and immune responses in HTX-offspring induced to EAE were the reduced suppressive capacity displayed by their T regulatory (T_reg_) lymphocytes (21). Therefore, it seems possible that the HTX-offspring can be more susceptible to suffering an enhanced autoimmune disease due to an imprint on T_reg_ lymphocytes and/or other immune cells. We hypothesized that gestational HTX could program the offspring’s immune system to develop more robust inflammatory responses against immune challenges. Recently, we reported that the HTX-offspring showed a significantly increased CD8^+^ T lymphocyte response during human metapneumovirus (hMPV) infection (22). Moreover, gestational hypothyroidism increased the offspring’s immune response during EAE (21) and against *Streptococcus pneumoniae* infection (8). It is also possible that gestational HTX could affect other types of cells or organs besides the immune system. Along these lines, there are molecules and factors that cells produce to protect themselves from oxidative damage, such as antioxidant enzymes like glutamate-cysteine ligase catalytic subunit (GCLC) (23), heme oxygenase-1 (HO-1) (24–27), NADPH quinone oxidoreductase 1 (NQO1) (28), and mucin-2 (MUC-2) (29). Therefore, we propose that HTX-offspring will be more susceptible to suffering immune-mediated diseases. The basis for this enhanced susceptibility consists of alterations in the immune system and the expression of cell-protective molecules in HTX-offspring, which promote inflammatory tissue damage. To test this hypothesis, the HTX-offspring and the offspring gestated in euthyroidism (Control-offspring) were induced to develop an ulcerative colitis-like disorder (UCLD). Ulcerative colitis (UC) is an immune-mediated inflammatory disorder of the colon characterized by chronic and continual mucosal inflammation (30). UC belongs to a group of pathologies classified as inflammatory bowel diseases (IBD), most of which have an unknown etiology (31). Patients who suffer from UC present abdominal pain, diarrhea, the presence of blood in the feces, and psychological distress (30). T lymphocytes, such as Th17 and T_reg_ lymphocytes, can contribute significantly to the pathogenesis of UC (32). In this study, UCLD was induced in the male HTX-offspring and the male Control-offspring by dextran sodium sulfate (DSS) administration for an acute period of 6 days (33). Pathophysiological scores were performed during UCLD induction. Histopathology of the colon was analyzed to determine whether gestational HTX can increase UCLD symptomatology offspring. To determine the mechanisms that could be affected in the HTX-offspring’s immune cells, the populations and contents of molecules with protective or inflammatory functions were measured in the colon tissue of the HTX-offspring.

## Materials and methods

### Animals

C57BL/6 were obtained from Jackson laboratory (Bar Harbor, ME, USA). Both breeders and the offspring were kept at the animal facility of Universidad Andres Bello. The mice were maintained with at 12-h light/dark cycle according to the Universidad Andrés Bello and Agencia Nacional de Investigación y Desarrollo (ANID) bioethics committee guidelines. All mice were supervised daily by a veterinarian.

### Maternal hypothyroxinemia induction

To perform breeding, two females and one male were placed in a cage, and the presence of a copulatory plug determined the next day’s pregnancy. That day was referred to as embryonic day 1 (E1). Then, each pregnant female was placed in a cage individually. Pregnant females were separated randomly into three groups: the first group, named Control, received tap drinking water during the whole pregnancy; the second group, named HTX, received 0.02% methimazole (MMI) (M8506, Sigma-Aldrich, St. Louis, MO, USA) in the tap drinking water from E10 to E14; and the third group, named as HTX+T4, received 0.02% of MMI in the tap drinking water and a daily subcutaneous injection of 25 μg/kg T_4_ in PBS from E10 to E14. The bottles containing MMI and T_4_ solution were prepared fresh every day and kept protected from light. Blood samples from the facial vein were obtained to analyze thyroid function at E14. tT_4_ was determined by chemiluminescence in a certified veterinary laboratory (LQCE). tT_3_ and TSH were assayed by ELISA (MBS704901, MyBioSource, San Diego, CA, USA). The offspring were weaned 30 days postnatal (P30) and separated by sex. Male offspring were used for UCLD induction at day P55 of age (weighing 18 to 25 g). The offspring was named after the mother treatment as the Control-offspring and the HTX-offspring. All mice were euthanized by isoflurane in 5% O_2_ inhalation according to AVMA guidelines (34).

### Induction and evaluation of ulcerative colitis-like disorder

UCLD was induced in male offspring both from Control and HTX by the administration of 2% DSS (42867, Sigma-Aldrich) in the drinking water for 6 days as previously described (35–37). The pathological score of UCLD was assessed daily during the whole treatment with DSS by measuring body weight, disease activity index (DAI) score, and fecal occult blood test (FOBT) score (33). The DAI score calculation corresponds to a qualitative visual analysis of two parameters: 1) stool consistency and 2) the presence of blood at the anus. A number is assigned to score the intensity of these symptoms (please see **Supplementary Table 1** for the score characterization).

### Histological analysis

Mice were euthanized on day 6 of UCLD induction, and the proximal, middle, and distal portions of the colon were obtained to analyze the histological signs of inflammation in these regions (38). For this purpose, tissues were fixed in 4% formaldehyde in PBS, embedded in paraffin, cut into 4–5 μm cross-sections, and stained with hematoxylin (100267, Newpath, Chile) and eosin (861006, Sigma-Aldrich). The stained sections were analyzed to obtain the histopathological score described previously (39) (see **Supplementary Table 2**). Per mice, three sections per portion of the colon were analyzed blindly under white-light microscopy (DM1000, Leica Microsystem, Germany).

### RT-qPCR analysis

Mice were euthanized on day 6 of UCLD induction, and colon samples were frozen at −80°C in RNA*later*® (R0901, Sigma-Aldrich). The samples were thawed in ice and then homogenized to further extract total RNA by the TRIzol® method (15596026, Thermo Fisher, Waltham, MA, USA). Total RNA was treated with DNAse I, quantified as described (7), and stored at −80°C. cDNA synthesis was performed with 1 µg of RNA using Affinity Script QPCR cDNA Synthesis Kit (600559, Agilent Technologies, Santa Clara, CA, USA) and oligo(dT) according to the manufacturer’s instructions. The primers used for the relative expression of genes encoding oxidative stress enzymes are shown in **Supplementary Table 3**. qPCR reactions were performed using Brilliant II SYBR Green QPCR Master Mix (600828, Agilent Technologies) following the manufacturer’s instructions, as described previously (7). Data were normalized against the housekeeping gene ribosomal protein S6 (*RPS6*) transcript. The data were analyzed using the 2^−ΔΔCT^ method and expressed as a fold change in gene expression relative to control.

### Flow cytometry analysis of colon immune cells

Mice from each experimental group were euthanized on day 6 of UCLD induction to obtain the colon to isolate the immune cells as previously described (40). Colon samples were dissected longitudinally, and feces were removed with cold PBS. The adipose tissue and Peyer patches were eliminated from the colon tissue. To eliminate epithelial cells, the minced colon tissue was incubated in Hank’s balanced salt solution (HBSS) + 2 mM of EDTA for 20 min at 37°C with constant agitation. Then, the solution was passed through a 70-μm cell strainer to separate cells from cellular debris (Bioscience Inc., San Diego, CA, USA). The obtained samples were digested with a solution of 1 mg/ml of collagenase VIII (C2139, Sigma-Aldrich), 1.25 mg/ml of collagenase D (11088858001, Sigma-Aldrich), 1 mg/ml of dispase (D4818, Sigma-Aldrich), and 20 μg/ml of DNase I (10104159001, Sigma-Aldrich) in HBSS–10% fetal bovine serum (FBS, A4766801, Thermo Fisher) for 20 min at 37°C with constant agitation (200 rpm). Cells were passed through a 70-μm cell strainer and centrifuged at 450×*g* for 5 min at 4°C, and the obtained pellet was resuspended in a PBS–5% FBS solution for flow cytometry analysis (41). First, cell suspensions were immunolabeled for extracellular markers. For that, cell suspensions were incubated in PEB buffer (PBS pH 7.4, 0.5% BSA, 2 mM EDTA) with the following antibodies at a 0.2-mg/ml concentration: anti-CD45-Bv421 (clone X54-5/7.1; BioLegend, San Diego, CA, USA), anti-CD3-FITC (clone RM4-5; BioLegend), anti-CD4-APC (clone RM4-5; BioLegend), anti-CD8-PE (clone RM4-5; BioLegend), anti-B220-PerCP 5.5 (clone RM4-5; BioLegend), anti-CD25-PE-Cy7 (clone PC61.5; BD Biosciences Inc., San Diego, CA, USA), anti-CD8a-PE/Cy7 (clone 53-6.7; BioLegend), anti-IA/IE-FITC (clone RM4-5; BioLegend), anti-CD11c-PE (clone RM4-5; BioLegend), anti-CD11b-PE-Cy7 (clone X54-5/7.1; BioLegend), anti-LY6C-PerCP 5.5 (clone RM4-5; BioLegend), and anti-LY6G-APC (clone RM4-5; BioLegend) for 1 h at room temperature (RT) in darkness. Then, the cells were fixed in 1% formaldehyde in PBS at RT. Afterward, the cells were incubated with 5 ng/ml of phorbol myristate acetate (PMA, 79346 Sigma-Aldrich) and 500 ng/ml of ionomycin (I0634, Sigma-Aldrich) for 3 h at 37°C. Next, the cells were immunolabeled for intracellular markers. For that, the cells were incubated in PEB buffer with the following antibodies: anti-Foxp3-PE (clone MF23-16s; BD Biosciences Inc.), anti-RorγT-Bv421 (clone Q31-378; BD Biosciences Inc.), and anti-IL-17a-APC-Cy7 (clone JES5-16E3; BD Biosciences Inc.) antibodies overnight at 4°C. Flow cytometry analysis was acquired using a FACSCanto II (BD Biosciences Inc.), and data were analyzed using FlowJo™ v10.8 Software (BD Life Sciences Inc.). Myeloid cells are shown as the percentage of neutrophils (CD11b^+^Ly6G^+^Ly6C^−^), monocytes (CD11b^+^Ly6G^−^Ly6C^+^), macrophages (CD11b^+^Ly6G^−^Ly6C^−^), and dendritic cells (DCs) (CD11b^−^CD11c^+^MHCII^+^) with respect to CD45^+^. The lymphoid cell population is presented as the percentage of B lymphocytes (CD3^-^B220^+^) and T cytotoxic (CD3^+^CD8^+^) concerning CD45. T regulatory cells (T_reg_) (CD25^+^Foxp3^+^) and Th17 cells (IL-17^+^RorγT^+^) were calculated as the percentage of CD3^+^. The gating strategy is shown in **Supplementary Figures 2**-**4**.

### Cytokine content from whole colon samples

The production of IL-17, IL-22, IFN-γ, TNF-α, and IL-10 was determined by ELISA in total protein extracts from the whole colon of all experimental groups. Briefly, total protein extraction was performed as previously described (42) by mechanical homogenization in 1 ml of radioimmunoprecipitation assay buffer (RIPA) (20 mM of Tris–HCl pH 7.5, 150 mM of NaCl, 1 mM of Na_2_EDTA, 1 mM of EGTA, 1% NP-40, 0.1% Triton X-100, 0.1% SDS) containing protease inhibitors (1 mM of PMSF, 0.1 mM of NaF, 200 mM of Na_3_VO_4_, 1 mM of leupeptin). Tissue homogenates were kept for 20 min on ice and then centrifuged at 18,000×*g* for 10 min. The supernatants were stored at −80°C until analysis. Total protein concentration was determined by the Pierce BCA protein assay kit (23221, Thermo Scientific) according to the manufacturer’s instructions. The contents of IL-17A (ELISA kit, 432501, BioLegend), IL-22 (436304, BioLegend), IFN-γ (430801, BioLegend), TNF-α (430901, BioLegend), and IL-10 (431411, BioLegend) were measured in 100 μg of total protein of colon samples according to the manufacturer’s instructions.

### Statistical analyses

Statistical analyses were performed using Prism software 9.1.0 (GraphPad Software, Inc., San Diego, CA, USA). Results are shown as mean ± SEM. Statistical differences for THs and TSH were tested using the Welch *t*-test. Multiple comparisons were performed using one-way ANOVA or two-way ANOVA, followed by Turkey’s *post-hoc* test or Kruskal–Wallis analysis with Dunn’s post-test for small samples. Statistical significance is indicated by *p <*0.05.

## Results

**Figure 1:**
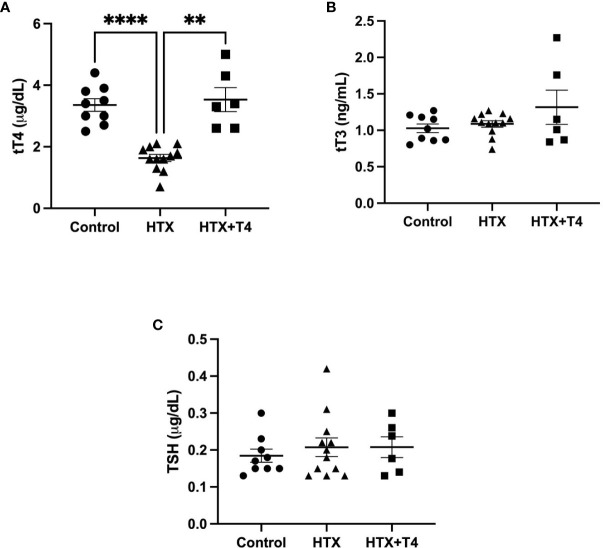
Methimazole treatment in pregnant mice induces gestational hypothyroxinemia (HTX). The contents of tT_4_, tT_3_, and thyroid-stimulating hormone (TSH) were measured on day 14 of pregnancy (E14) in pregnant mice treated with tap drinking water (Control), MMI (HTX), or MMI and T_4_ (HTX+T4) during E10–E14. The contents of tT_4_, tT_3_, and TSH are plotted in **(A–C)**, respectively. The values in the graphs are presented as mean ± SEM (Student’s *t*-test). Statistical significance is indicated as **p ≤ 0.01 and ****p < 0.0001. Pregnant dams: tT_3_: Ctrl *N* = 9, HTX *N* = 12, HTX+T4 *N* = 6. tT_4_: Ctrl *N* = 9, HTX *N* = 12, HTX+T4 *N* = 6. TSH: Ctrl *N* = 9, HTX *N* = 12, HTX+T4 *N* = 6.

Gestational HTX was induced in pregnant mice by the administration of 2% MMI in drinking water from day 10 to 14 of pregnancy (E10–E14) (see *Materials and methods*), and the name of this experimental group is HTX. MMI is an inhibitor of thyroid peroxidase (TPO) the enzyme that synthesizes THs in the thyroid gland (43). It has been widely shown that the short administration of MMI during gestation produces transient HTX (7, 11, 21, 44, 45). To ensure that the UCLD symptoms in the offspring gestated in HTX (HTX-offspring) are due to T_4_ reduction during pregnancy and not to a secondary effect of MMI. A second experimental group was included that reestablished T_4_ levels during MMI treatment, and the name of this group is HTX+T4. To confirm that gestational HTX was induced in pregnant mice treated with MMI, tT_4_, tT_3_, and TSH were measured from the blood samples taken from all pregnant mice on day 14 of pregnancy. Thyroid hormones and TSH levels are plotted in **Figure 1**. The HTX group that received 2% MMI showed a reduction only in tT_4_ and normal levels of tT_3_ and TSH were observed in the serum (**Figures 1A–C**). Control and HTX+T4 pregnant mice showed similar levels of tT_4_, tT_3_, and TSH on day 14 of pregnancy. These results indicated that mice treated only with MMI suffered gestational HTX. For HTX+T4 that also received MMI and T_4_, the levels of T_4_ were restored to normal levels.

### UCLD symptoms are accelerated and enhanced in the male offspring gestated in HTX as compared with the offspring gestated under euthyroidism

**Figure 2:**
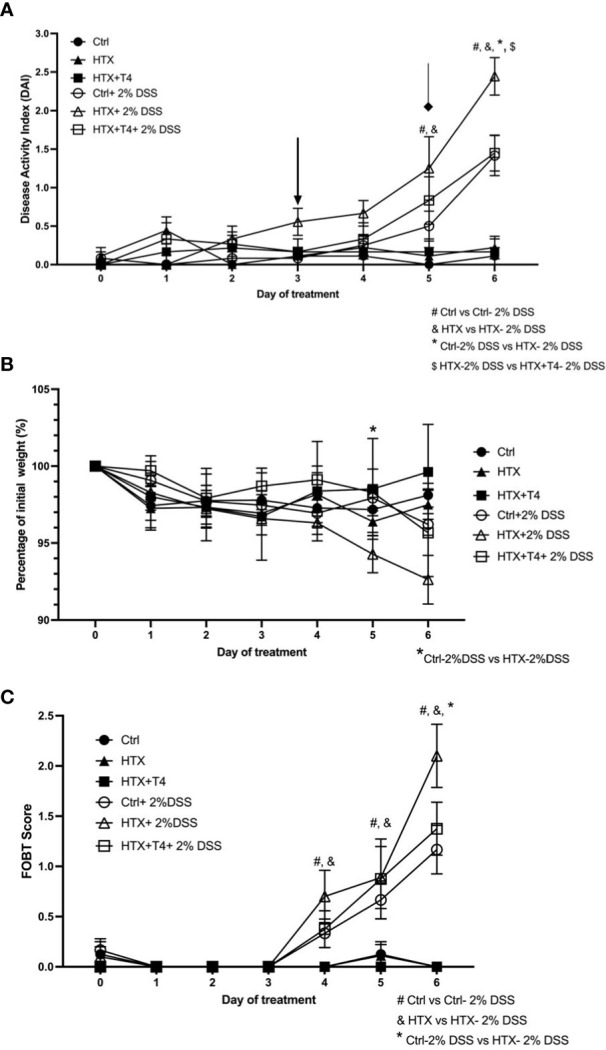
Accelerated and enhanced pathological UCLD symptoms were observed in male HTX-offspring treated with 2% DSS for 6 days. Male HTX-offspring, Control-offspring, and HTX+T4-offspring were orally treated with 2% dextran sodium sulfate (DSS) for 6 days to induce acute ulcerative colitis-like disorder (UCLD). **(A)** The graph shows the analysis of the disease activity index (DAI). The arrow shows the early appearance of UCLD signs in the HTX-offspring. The diamond indicates the appearance of UCLD signs for the Control-offspring and HTX+T4-offspring. **(B)** The body weight percentage was registered daily for each experimental offspring induced or not with UCLD disease. **(C)** The presence of blood in the feces was determined by the fecal occult blood test (FOBT) analysis for each experimental group. The values are shown as mean ± SEM (two-way ANOVA and Tukey’s post-test). Statistical significance is indicated as # for the Ctrl vs. the Ctrl+2% DSS group, & the HTX vs. the HTX+2% DSS group, and * for the Ctrl+2% DSS vs. the HTX+2% DSS group when *p* < 0.05. Ctrl *N* = 15, HTX *N* = 17, Ctrl+2%DSS *N* = 17, HTX+2%DSS *N* = 17, HTX+T4 *N* = 7, and HTX+T4 + 2%DSS *N* = 8.

The male progenies gestated in HTX, Control, and HTX+T4 were induced on postnatal day 55 (P55) with acute UCLD by the administration of 2% DSS in drinking water for 6 days (see *Materials and methods*). DSS is a sulfated polysaccharide widely used in animal models to induce UCLD (33, 39). All experimental groups treated or not with DSS were monitored daily for 6 days. UCLD pathological scores were registered using the previously described scale (33, 38). The symptoms of UCLD in the HTX-offspring started on day 3 of DSS administration (see black arrow in **Figure 2A**) as compared with the Control-offspring when symptoms appeared on day 5 after DSS treatment (see diamond arrow in **Figure 2A**). The HTX-offspring showed significantly higher pathological scores on day 5 and day 6 after DSS administration as compared with the Control-offspring and on day 6 as compared with the HTX+T4-offspring (**Figure 2A**). As expected, all experimental groups not treated with DSS did not display pathological scores (**Figure 2A**). Weight loss was measured during 6 days after DSS treatment, and all experimental groups showed equivalent body weight loss during early time points of the experiments (**Figure 2B**). Only on day 5 of DSS treatment, the HTX-offspring showed a significant loss of weight as compared with the Control-offspring and on day 4 as compared with the HTX+T4-offspring. The FOBT was used to analyze the presence of blood in the feces (**Figure 2C**). All experimental groups treated with DSS showed blood in the feces on day 4; however, it was significantly higher in the HTX-offspring on day 6 as compared with the Control-offspring and HTX+T4-offspring (**Figure 2C**).

### Male HTX-offspring showed UCLD histopathological scores at the proximal, medial, and distal colon tissues

**Figure 3:**
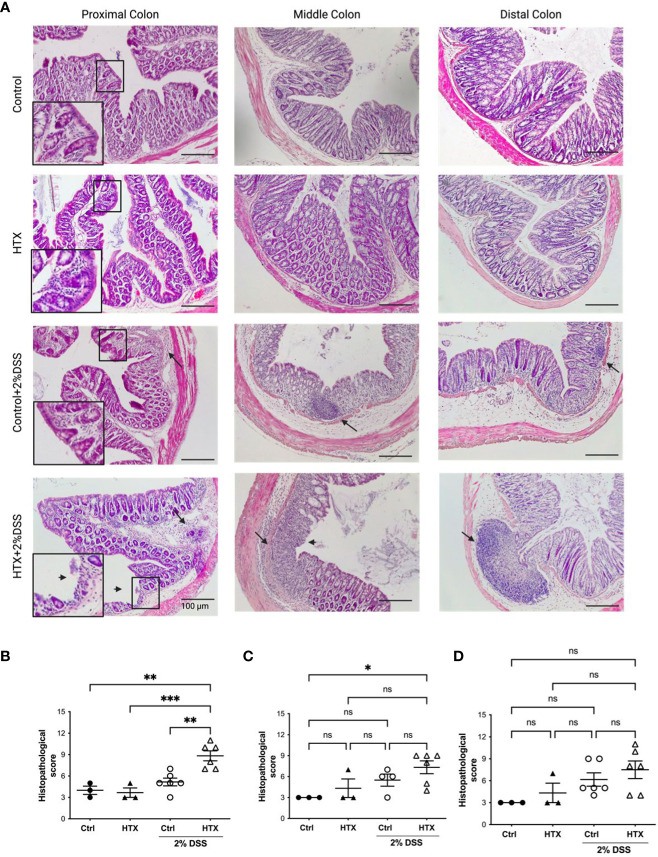
Male HTX-offspring with UCLD has inflammation signs at the proximal, middle, and distal colon. The HTX-offspring and Control-offspring were orally treated with 2% DSS for 6 days to induce UCLD. On day 6, mice were euthanized, and the colon was isolated and stained with hematoxylin and eosin to perform histopathological analysis. **(A)** Images were taken from the proximal, middle, and distal colon of the HTX-offspring and Control-offspring induced or not with UCLD. A digital zoom of the mucosa appears to show signs of tissue damage. The arrow (→) shows the inflammation area or lymphatic nodules, and the arrowhead (➤) shows mucosa erosion. Histopathological score analyses of the proximal, middle, and distal colon regions were plotted in **(B–D)**, respectively. The values in the graphs represent mean ± SEM (one-way ANOVA and Tukey’s post-test). Statistical significance is indicated as *p ≤ 0.05, **p ≤ 0.01, *** *p *< 0.001 and non significant (ns). **(B)** Ctrl *N* = 3, HTX *N* = 3, Ctrl+2%DSS *N* = 6, and HTX+2%DSS *N* = 6. **(C)** Ctrl *N* = 3, HTX *N* = 3, Ctrl+2%DSS *N* = 4, and HTX+2%DSS *N* = 6. **(D)** Ctrl *N* = 3, HTX *N* = 3, Ctrl+2%DSS *N* = 6, and HTX+2%DSS *N* = 6.

UC is characterized by histopathological signs of inflammation in the mucosa of the colon mainly at the distal and medial regions (38). Coronal sections of the proximal, middle, and distal colon from the HTX-offspring and Control-offspring induced or not with UCLD were stained with hematoxylin and eosin (see *Materials and methods*). Representative images for these sections of all experimental groups are shown in **Figure 3A**. The average of the inflammatory scores for the proximal, middle, and distal colon is shown in **Figures 3B–D**, respectively, and was calculated based on a scoring system summarized in **Supplementary Table 2** (40). The histopathological scores of the proximal, middle, and distal colon for the HTX-offspring and Control-offspring without UCLD induction were not zero (**Figures 3B–D** and see **Supplementary Figure 1**) because minimal cell infiltration was detected. This cell infiltration was greater for the HTX-offspring, and tissue distortion was also observed in this progeny. The HTX-offspring and Control-offspring induced with UCLD showed higher histopathological scores at the proximal, middle, and distal portions of the colon than the HTX-offspring and Control-offspring non-induced with UCLD. Some of these features were distortion of the tissue, erosions, and extensive inflammatory infiltration affecting different layers of the colon. In **Figure 3A**, lymphatic nodules in the Control-offspring and HTX-offspring with UCLD were observed, showing that, in the HTX-offspring, the lymphatic nodule is bigger reaching the submucosa layer. Even though the histopathological scores of the middle and distal portions of the colon were not significantly different between the HTX-offspring and Control-offspring induced with UCLD (**Figures 3C, D**, respectively), the histopathological score for the proximal colon was significantly higher for the HTX-offspring with UCLD compared to the Control-offspring with UCLD (**Figure 3B**).

### Lower content of GCLC and MUC-2 mRNA in the colon tissue from male HTX-offspring

**Figure 4:**
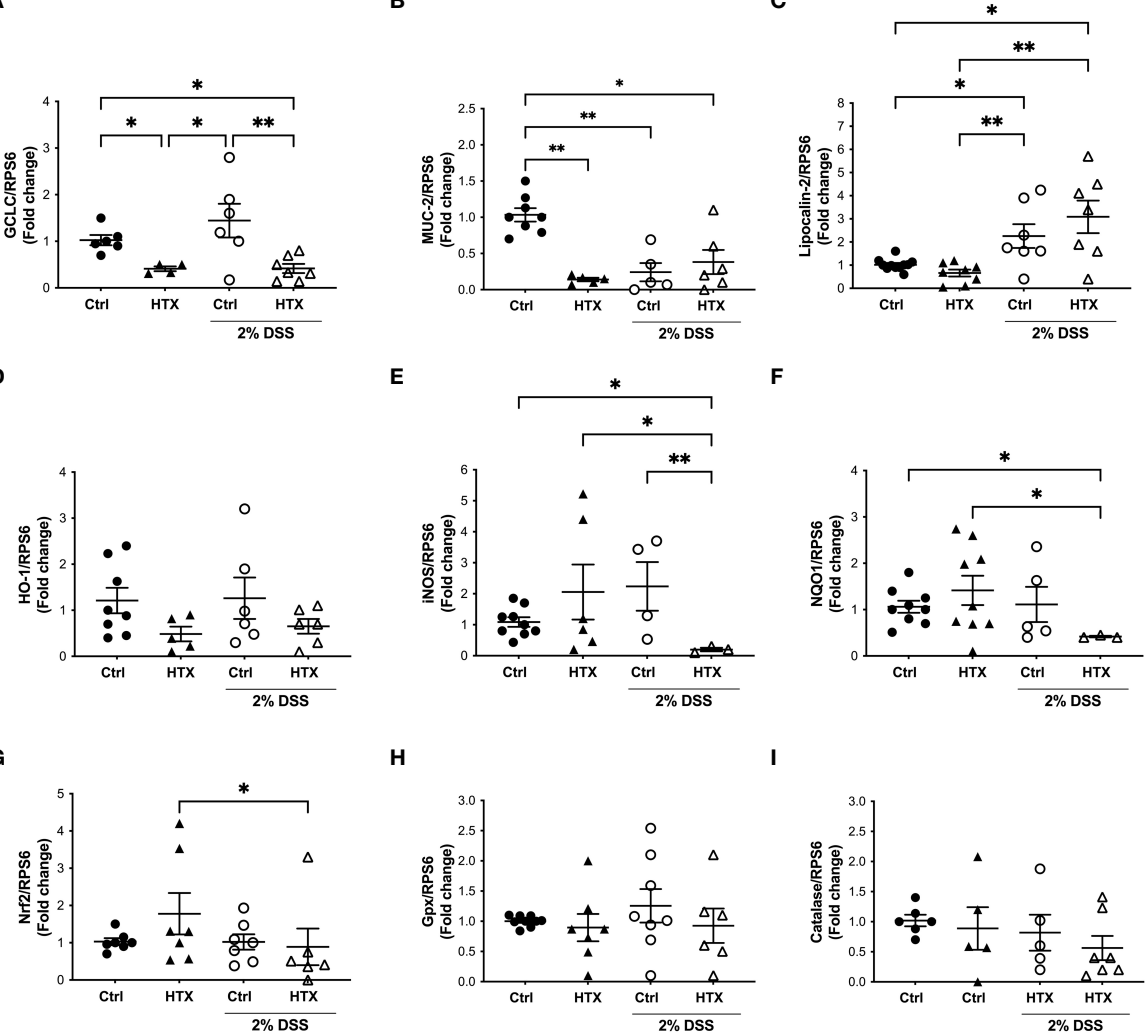
GCLC or MUC-2 mRNA levels were reduced in male HTX-offspring. The HTX-offspring and Control-offspring were orally treated with 2% dextran sodium sulfate (DSS) for 6 days to induce ulcerative colitis like disorder (UCLD). On day 6, mice were euthanized, and mRNA was isolated from the colon tissue to be analyzed by RT-qPCR. The mRNA levels were plotted as arbitrary units (a.u.) concerning the mRNA expression for the housekeeping gene *RPS6* used as a normalizer in the figures. **(A)** GCLC; **(B)** MUC-2; **(C)** Lipocalin-2; **(D)** HO-1; **(E)** iNOS; **(F)** NQO1; **(G)** NRF2; **(H)** Gpx; and **(I)** catalase. The values in the graphs are presented as mean ± SEM (Kruskal–Wallis and Dunn’s post-test). Statistical significance is indicated as *p ≤ 0.05, **p ≤ 0.01. **(A)** Ctrl *N* = 6, HTX *N* = 4, Ctrl+2%DSS *N* = 6, and HTX+2%DSS *N* = 7. **(B)** Ctrl *N* = 8, HTX *N* = 5, Ctrl+2%DSS *N* = 5, and HTX+2%DSS *N* = 6. **(C)** Ctrl *N* = 11, HTX *N* = 8, Ctrl+2%DSS *N* = 7, and HTX+2%DSS *N* = 7. **(D)** Ctrl *N* = 8, HTX *N* = 5, Ctrl+2%DSS *N* = 6, and HTX+2%DSS *N* = 6. **(E)** Ctrl *N* = 9, HTX *N* = 6, Ctrl+2%DSS *N* = 4, and HTX+2%DSS *N* = 3. **(F)** Ctrl *N* = 9, HTX *N* = 9, Ctrl+2%DSS *N* = 5, and HTX+2%DSS *N* = 3. **(G)** Ctrl *N* = 7, HTX *N* = 7, Ctrl+2%DSS *N* = 7, and HTX+2%DSS *N* = 6. **(H)** Ctrl *N* = 8, HTX *N* = 7, Ctrl+2%DSS *N* = 8, and HTX+2%DSS *N* = 6. **(I)** Ctrl *N* = 6, HTX *N* = 5, Ctrl+2%DSS *N* = 5, and HTX+2%DSS *N* = 7.

Previous studies showed that oxidative stress increases during the inflammatory response of UC (46). Even though, the intestine counts with antioxidants and molecules that have protective roles to counteract inflammation, a reduced expression of these agents could contribute to the enhanced inflammatory UCLD (47). Therefore, mRNA relative expression encoding for antioxidant or oxidative stress proteins was measured in the colon of the HTX-offspring and Control-offspring with or without UCLD (**Figure 4**). The mRNA content of GCLC, the first rate-limiting enzyme of glutathione synthesis, was quantified in the colon samples for all experimental groups (23). Significantly lower levels of *GCLC* mRNA were found in the HTX-offspring as compared with the Control-offspring with or without UCLD induction (**Figure 4A**). Interestingly, we observed an increase, although not significant, in the expression of *GCLC* in the colon samples from the Control-offspring induced with UCLD as compared with those not induced with UCLD. On the contrary, no increase of *GCLC* mRNA was detected in the HTX-offspring induced with UCLD as compared to mice in which UCLD was not induced. The mRNA content of MUC-2, an essential protein for normal intestinal barrier function, was measured in the colon samples derived from all experimental groups (29). The HTX-offspring not induced with UCLD showed a significantly lower expression of *MUC-2* mRNA levels as compared with the Control-offspring (**Figure 4B**). However, after UCLD induction, the Control-offspring showed reduced *MUC-2* mRNA levels equivalent to the HTX-offspring (**Figure 4B**). The observed reduction of *MUC-2* mRNA is consistent with the barrier damage reported for UC murine models (48). Lipocalin-2 is a bacteriostatic protein (49) whose expression has been shown to be increased in patients with active IBD (49) and in murine models for UC (50). An increase in *Lipocalin-2* expression was observed in both the Control-offspring and HTX-offspring suffering from UCLD, with no significant differences between these two experimental groups (**Figure 4C**). This result was consistent with previous observations in the murine model for UCLD (50). HO-1 is an enzyme that reduces inflammation and plays a modulatory role during intestinal inflammation (24). No differences were observed in the expression of *HO-1* mRNA between any experimental group (**Figure 4D**). Even though there were no significant differences among these groups, there is a trend for reduced expression of *HO-1* mRNA in the HTX-offspring (**Figure 4D**). The expression of inducible nitric oxide synthetase (iNOS) has been reported to increase in UC patients (51). Given that iNOS synthesizes nitric oxide (NO), a high expression of this enzyme has been associated with tissue damage (52). A significant decrease in the relative expression of *iNOS* was found in the HTX-offspring suffering from UCLD when compared with the HTX-offspring without UCLD induction (**Figure 4E**) and when compared with the Control-offspring with or without UCLD induction (**Figure 4E**). No significant differences were observed for the mRNA relative expression of *NQO1* (**Figure 4F**), *Nrf2* (**Figure 4G**), *Gpx* (**Figure 4H**), and *catalase* (**Figure 4I**) between the HTX-offspring and Control-offspring suffering from UCLD. A significant reduction in the levels of NQO1 mRNA of the HTX-offspring induced with UCLD compared with the HTX-offspring without UCLD induction and the Control-offspring without UCLD induction was found (**Figure 4F**). The level of Nrf2 mRNA was also reduced in the HTX-offspring induced with UCLD as compared with the HTX-offspring without UCLD induction (**Figure 4G**).

### The population of Th17 lymphocytes is higher in the colon of male HTX-offspring with or without UCLD

**Figure 5:**
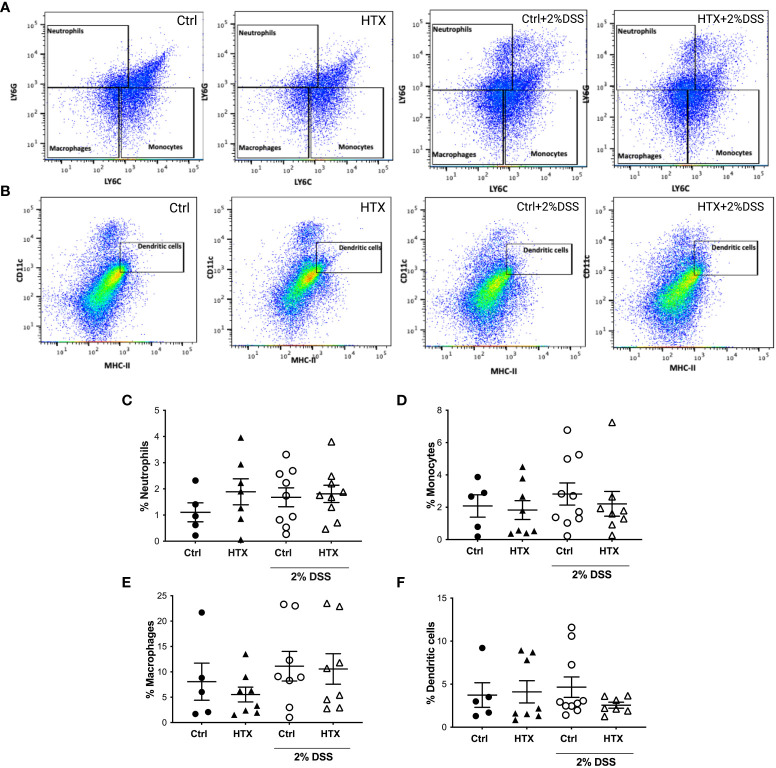
The percentage of myeloid cells in the colon of male HTX-offspring was similar to the Control-offspring induced or not with UCLD. The HTX-offspring and Control-offspring were orally treated with 2% dextran sodium sulfate (DSS) for 8 days to induce ulcerative colitis-like disorder (UCLD). On day 6, mice were euthanized, and the colon was isolated to obtain immune cells that were incubated with specific antibodies to detect myeloid cells by flow cytometry analysis. **(A)** Representative dot-plots for neutrophils, monocytes, and macrophages are shown for each experimental group induced or not to develop UCLD. **(B)** Representative dot-plots for DC detection are shown for each experimental group induced to develop UCLD. The graphs with the percentage of neutrophils, monocytes, macrophages, and DCs for each experimental group are shown in **(C–F)**, respectively. No significant differences between groups were observed. The values in the graphs are presented as mean ± SEM (Kruskal–Wallis and Dunn’s post-test). **(C)** Ctrl *N* = 5, HTX *N* = 7, Ctrl+2%DSS *N* = 9, and HTX+2%DSS *N* = 9. **(D)** Ctrl *N* = 5, HTX *N* = 8, Ctrl+2%DSS *N* = 10, and HTX+2%DSS *N* = 8. **(E)** Ctrl *N* = 5, HTX *N* = 8, Ctrl+2%DSS *N* = 8, and HTX+2%DSS *N* = 8. **(F)** Ctrl *N* = 5, HTX *N* = 8, Ctrl+2%DSS *N* = 10, and HTX+2%DSS *N* = 7.

**Figure 6:**
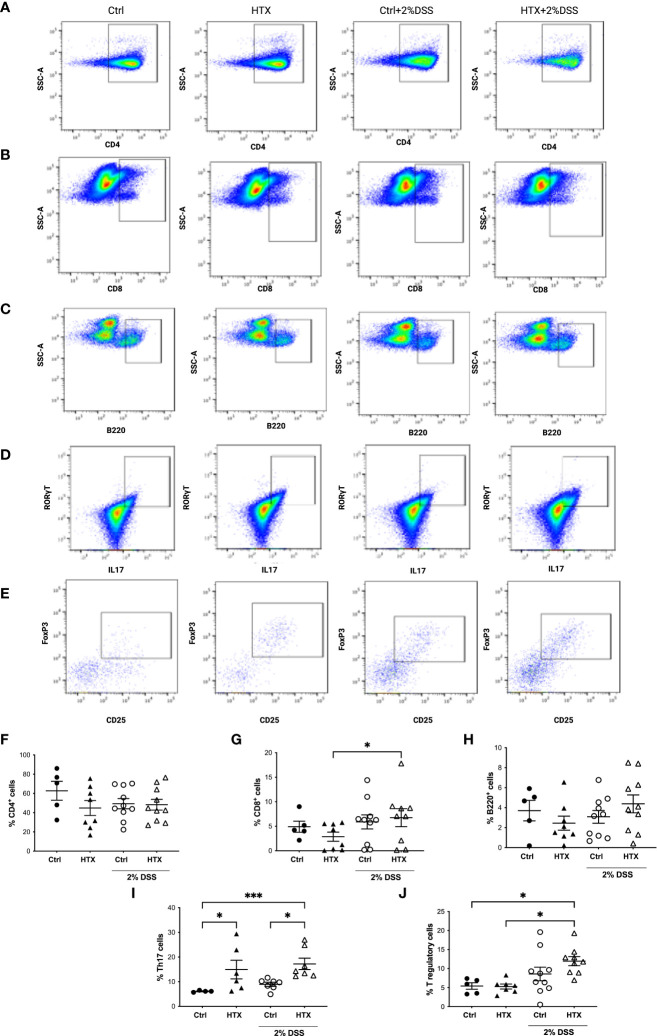
Increased percentage of Th17 lymphocytes in the colon of male HTX-offspring induced or not to develop UCLD. The HTX-offspring and Control-offspring were orally treated or not with 2% dextran sodium sulfate (DSS) for 6 days to induce ulcerative colitis-like disorder (UCLD). On day 6, mice were euthanized, and the colon was isolated to obtain immune cells incubated with antibodies to detect lymphocyte populations analyzed by flow cytometry. Representative dot plots for CD4^+^ T lymphocytes **(A)**, CD8^+^ T lymphocytes **(B)**, B lymphocytes **(C)**, Th17 lymphocytes **(D)**, and T_reg_ lymphocytes **(E)** for each experimental group. The graphs with the percentage of CD4^+^ T, CD8^+^ T, B, Th17, and T_reg_ lymphocytes are shown in **(F–J)**, respectively. The values in the graphs are presented as mean ± S.E.M. Kruskal-Wallis and Dunn´s post-test. Statistical significance is indicated as *p ≤ 0.05, *** *p *< 0.001. **(F)** Ctrl *N* = 5, HTX *N* = 8, Ctrl+2%DSS *N* = 10, and HTX+2%DSS *N* = 10. **(G)** Ctrl *N* = 5, HTX *N* = 8, Ctrl+2%DSS *N* = 10, and HTX+2%DSS *N* = 9. **(H)** Ctrl *N* = 5, HTX *N* = 8, Ctrl+2%DSS *N* = 10, and HTX+2%DSS *N* = 10. **(I)** Ctrl *N* = 4, HTX *N* = 6, Ctrl+2%DSS *N* = 7, and HTX+2%DSS *N* = 7. **(J)** Ctrl N=5, HTX N=7, Ctrl+2%DSS *N* = 10, and HTX+2%DSS *N* = 9.

The immune system plays a pivotal role in the development and outcome of UC pathology (53). Therefore, myeloid and lymphoid cell populations were quantified by flow cytometry from the colon of the HTX-offspring and Control-offspring without induction of UCLD and after 6 days of UCLD induction (47). Representative dot plots for neutrophils, monocytes, and macrophages are shown in **Figure 5A** and that for DCs is shown in **Figure 5B**. The gating strategy is indicated in **Supplementary Figures 2**-**4**. The graphs of flow cytometry analyses of all experimental groups were plotted in **Figure 5C** for neutrophils, in **Figure 5D** for monocytes, and in **Figure 5E** for macrophages. A similar percentage of neutrophils (**Figure 5C**), monocytes (**Figure 5D**), macrophages (**Figure 5E**), and DCs (**Figure 5F**) was observed for all experimental offspring induced or not with UCLD. A subtle increase was observed in the Control-offspring and HTX-offspring suffering from UCLD as compared with the Control-offspring not induced with UCLD. Even though no significant differences were observed, some individuals gestated in HTX and not suffering from UCLD showed more neutrophils in the colon as compared with the Control-offspring with no UCLD. Flow cytometry analyses of various lymphocyte populations from the colon for each experimental group are shown in **Figure 6**. Representative dot-plots for CD4^+^ T lymphocytes, CD8^+^ T lymphocytes, B lymphocytes, Th17 lymphocytes, and T regulatory (T_reg_) lymphocytes are shown in **Figures 6A–E**, respectively. The percentage of CD4^+^ T lymphocytes was similar among all offspring (**Figure 6F**). A significant increase of CD8^+^ T lymphocytes was found in the HTX-offspring induced with UCLD as compared with the HTX-offspring not induced with UCLD (**Figure 6G**). However, the percentage of CD8^+^ T lymphocytes was similar between the HTX-offspring and the Control-offspring induced with UCLD. The percentage of B lymphocytes was similar among the four experimental groups induced or not with UCLD (**Figure 6H**). The Th17 lymphocyte population was significantly increased in the colon of both HTX-offspring induced or not with UCLD compared with the Control-offspring induced or not with UCLD, respectively (**Figure 6I**). Not statistically significant differences were observed in any cytokine analyzed and between any experimental group. The T_reg_ lymphocyte percentage was similar between all experimental groups induced or not with UCLD (**Figure 6J**).

### The levels of inflammatory and anti-inflammatory cytokines remained similar in the colon tissue of the HTX-offspring and Control-offspring with or without 6 days of UCLD induction

**Figure 7:**
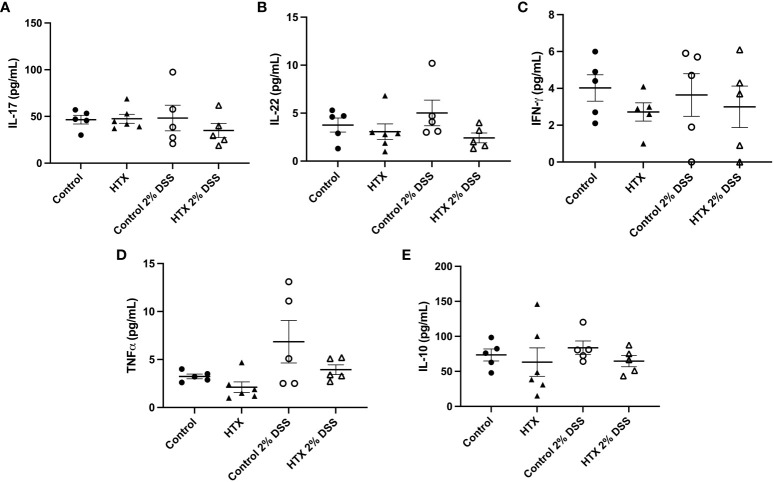
Content of IL-17, IL-22, IFN-γ TNF-α and IL-10 was similar between HTX- and Control-offspring. The HTX-offspring and Control-offspring were orally treated with 2% dextran sodium sulfate (DSS) for 6 days to induce ulcerative colitis-like disorder (UCLD). On day 6, mice were euthanized, and the colon was used to obtain a total protein sample for cytokine analysis by ELISA. The content was plotted in the following graphs: **(A)** IL-17, **(B)** IL-22, **(C)** IFN-γ, **(D)** TNF-α, and **(E)** IL-10. No significant differences were observed between the experimental groups. **(A)** Ctrl *N* = 5, HTX *N* = 6, Ctrl+2%DSS *N* = 5, and HTX+2%DSS *N* = 5. **(B)** Ctrl *N* = 5, HTX *N* = 6, Ctrl+2%DSS *N* = 5, and HTX+2%DSS *N* = 5. **(C)** Ctrl *N* = 5, HTX *N* = 5, Ctrl+2%DSS *N* = 5, and HTX+2%DSS *N* = 5. **(D)** Ctrl *N* = 5, HTX *N* = 6, Ctrl+2%DSS *N* = 5, and HTX+2%DSS *N* = 5. **(E)** Ctrl *N* = 5, HTX *N* = 6, Ctrl+2%DSS *N* = 5, and HTX+2%DSS *N* = 5. The values in the graphs are presented as mean ± SEM (one-way ANOVA and Tukey’s post-test).

Cytokine secretion at the colon tissue can contribute to the development and intensity of UCLD (54, 55); therefore, the content of IL-17, IL-22, IFN-γ, TNF-α, and IL-10 was measured by ELISA in the colon samples of all experimental groups (**Figure 7**). Not statistically significant differences were observed in any cytokine analyzed and between any experimental group.

## Discussion

This study shows evidence that male HTX-offspring develops earlier and increased pathological symptoms of UCLD compared with the male Control-offspring (**Figure 2**). These data agree with previous findings showing that the HTX-offspring suffers early and strong symptoms of EAE (21). The fact that the DAI score of the HTX+T4-offspring was similar to the Control-offspring indicated that the earlier onset and intense symptoms of UCLD of the HTX-offspring are consequences of low T_4_ during pregnancy and not due to other secondary effects of MMI treatment (**Figure 2**). The UCLD animal model used in this study can be considered as an acute model of UCLD given that the treatment with DSS was only for 6 days, and the analysis of inflammation on day 6 corresponds to the beginning of UC symptoms (33). The reason why we chose an acute model was based on our main question regarding whether gestational HTX can be a risk factor for increasing the susceptibility of earlier and strong immune-mediated diseases like UCLD. It has been documented that there is an association between UCLD pathological scores and colon tissue damage in the DSS model for UC-IBD (56). In this study, the HTX-offspring and Control-offspring treated with DSS presented scores of UCLD on day 3 and day 5 of DSS treatment, respectively. These UCLD pathological scores were confirmed by the histopathological analysis on day 6, by observing signs of tissue damage, such as distortion, erosion, cryptitis, and cellular infiltration at the proximal, middle, and distal colon in all experimental animal groups induced with UCLD. The histopathological analysis also reveals that both the Control-offspring and HTX-offspring without induction of UCLD have a histopathological score greater than zero and these were due to cell infiltration at the mucosa (see **Figure 3**, **Supplementary Figure 1**). The histopathological score significantly increased only at the proximal colon sections in the HTX-offspring induced with UCLD compared with the non-induced UCLD groups and the Control-offspring induced with UCLD (**Figure 3B**). This result was unexpected given that it has been reported that the signs of damage in humans and mice suffering from UCLD start at the distal portion of the colon and continue ascending through the middle colon until the proximal region (57, 58). In our results, the histopathological scores at the distal and middle portions of the colon were similar between the Control-offspring and HTX-offspring with UCLD (**Figures 3C, D**). However, the histopathological score at the proximal colon was significantly higher for the HTX-offspring induced with UCLD as compared with the Control-offspring induced with UCLD (**Figure 3B**). These results suggest that the HTX-offspring could be more sensitive in developing inflammation, given that by inducing acute UCLD their proximal colon showed more damage compared with the proximal colon of the Control-offspring. In this work, we found evidence that the protective mechanisms at the colon can be impaired in the HTX-offspring. This is the case for *MUC-2* mRNA levels that were found significantly reduced in the HTX-offspring without UCLD induction as compared with the Control-offspring without UCLD induction (**Figure 4B**). *MUC-2* encodes for a glycoprotein that is essential for the mucus to serve as the first barrier for protecting the intestinal epithelium from direct contact with the microbiota (29). Also, significantly lower levels of GCLC mRNA were found in the colon of the HTX-offspring with or without UCLD compared with the Control-offspring with or without UCLD, respectively (**Figure 4A**). GCLC mRNA encodes for an antioxidant enzyme that protects the colon from tissue damage (59, 60). The low levels of GCLC and MUC-2 mRNAs in the HTX-offspring support the notion that they could have a reduced capacity to protect the intestinal barrier against an insult. Moreover, the HTX-offspring induced with UCLD showed significantly lower levels of *iNOS* mRNA compared with the Control-offspring induced with UCLD (**Figure 4E**), suggesting that the HTX-offspring could have low levels of NO after acute UCLD induction (61). Several reports in the literature support that NO has a protective role at the beginning of UC and it becomes harmful at advanced stages of the disease (61–63). Therefore, based on the pathological score of UCLD, we suggest that the low expression of *iNOS* in the HTX-offspring induced with acute UCLD would produce less NO. Low levels of NO in the HTX-offspring will leave the colon more sensitive to tissue damage. The levels of *Lipocalin-2* mRNA were higher in the HTX-offspring and Control-offspring induced with UCLD compared with the HTX-offspring and Control-offspring not induced with UCLD, respectively (**Figure 4C**). A correlation matrix analysis showed that the content of Lipocalin-2 correlated with more intense UCLD (**Supplementary Figure 5**). These results support that Lipocalin-2 secreted by macrophages, neutrophils, and parenchymal cells and its increased secretion during UC could serve as predictor markers of this disease (64).

The symptoms of UCLD started 3 days after DSS treatment in the HTX-offspring, which was significantly earlier as compared with 5 days for the Control-offspring (**Figure 2**). These observations align with previous evidence that the HTX-offspring showed increased susceptibility to suffering enhanced EAE symptoms (21). Therefore, these findings suggest that gestational HTX could imprint the progeny to be prone to exacerbation of immune and inflammatory responses. This notion is supported by the observations made with the HTX-offspring challenged with inflammatory disease models for EAE (21) or UCLD (**Figure 2**), as well as after viral (22) or bacterial infections (8). In addition, offspring gestated in hypothyroidism and infected with *Streptococcus pneumoniae* developed stronger immune responses leading to protection against bacterial infection and dissemination, as well as increased host survival (8). Furthermore, although the HTX-offspring infected with the hMPV showed reduced viral loads, they displayed higher pathological scores due to an exacerbated immune response (22). We believe that the identification of the cellular and molecular mechanisms responsible for the immune system alterations observed in the HTX-offspring will contribute to molecular targets to further study the imprinting of this condition at the epigenetic level. Both innate and adaptive immune responses play important roles during autoimmune disease and infection (27, 65–67). For that reason, in this study, myeloid and lymphoid cell populations resident in the colon were analyzed by flow cytometry (**Figures 5**, **6**, respectively). It has been shown that Th1, Th17, and T_reg_ lymphocytes are the primary T cells involved in UCLD. Therefore, Th17, CD4^+^ T, CD8^+^ T, T_reg_, and B lymphocyte cell populations were analyzed in the colon samples from all experimental mice (**Figure 6**). Even though we did not observe statistically significant differences in CD4^+^ T and CD8^+^ T lymphocytes between the HTX-offspring and the Control-offspring, there was a significant increase in the percentage of CD8^+^ T lymphocytes for the HTX-offspring induced with UCLD compared with the HTX-offspring without UCLD (**Figure 6G**). Consistent with this observation, children suffering from UC show higher numbers of CD8^+^ T lymphocytes in their blood samples, which is associated with increased colon injury, such as ulcer formation (68). Therefore, it would be important to further study the contribution of CD8^+^ T lymphocytes in the colon, blood, LMN, and spleen in the HTX-offspring and a possible association with increased UCLD pathological scores. Regarding T_reg_ lymphocytes, no significant differences were observed between the HTX-offspring and the Control-offspring suffering from UCLD (**Figure 6J**). However, T_reg_ lymphocytes showed a significant increase in the HTX-offspring suffering from UCLD, as compared with those not treated with DSS (**Figure 6J**). This result was unexpected given that the percentage of T_reg_ from the spleen did not change in the HTX-offspring induced with EAE (21). However, these cells showed reduced suppressive capacity as compared with T*_reg_* from the Control-offspring induced to develop EAE (21). Even though the percentage of T_reg_ lymphocytes was higher in the colon of the HTX-offspring induced with UCLD, the content of IL-10 was similar among these groups (**Figure 7E**). The suppressive capacity of T_reg_ from the spleen or colon was not analyzed in these experimental animal groups, and further studies should evaluate the functional capacity of these cells. Noteworthy was the observation that gestational HTX increases the percentage of Th17 lymphocyte population in their offspring with or without UCLD when they were compared with the Control-offspring with or without UCLD, respectively (**Figure 6I**). Thus, this result will suggest that the great number of Th17 lymphocytes in the HTX-offspring will contribute to the early onset and/or the high pathological score of UCLD, given that it has been reported that the immune response of Th17 lymphocytes is one of the principal pathological mechanisms involved in UC (69). Th17 cells in an inflammatory environment can increase the proportion of Th1 lymphocytes and the secretion of IFN-γ by these cells, and probably by this mechanism, Th17 cells augment the intestinal injury and the disruption of the intestinal barrier in UC (70). This mechanism is unlikely to occur in the HTX-offspring given that the HTX-offspring with or without UCLD showed similar levels of IFN-γ compared with the Control-offspring (**Figure 7C**). Moreover, the content of IL-17 and IL-22 in the HTX-offspring induced with UCLD was similar to the Control-offspring induced with UCLD (**Figures 7A, B**, respectively). These results were unexpected given that there are more Th17 lymphocytes in the colon of the HTX-offspring than in the Control-offspring. A possible explanation for this observation is that Th17 cells from the HTX-offspring are not completely functional and secrete less IL-17 and IL-22. Under this scenario, we think that the HTX-offspring will need to augment the number of Th17 cells aiming to increase the production of these cytokines to protect the intestine. In fact, it has been shown that Th17 cells and these cytokines are important for a healthy homeostasis environment in the intestine to improve defense against extracellular pathogens by recruiting neutrophils and increasing intestinal barrier integrity (70, 71). Furthermore, that it has been described in the literature that Th17 lymphocytes can be anti-inflammatory or inflammatory and that the type of microbiota present at the intestinal lumen could play a role in the function of Th17 cells (72).

The HTX-offspring had a similar percentage of B lymphocytes in the colon (**Figure 6H**). It has been reported that UCLD induced by DSS increases the number of myeloid cells, such as DCs, neutrophils, monocytes, and macrophages (73, 74). The percentage of neutrophils, monocytes, macrophages, and dendritic cells was similar in all experimental groups (**Figure 5**). It is possible that the low levels of DSS used in this study were insufficient to significantly increase the number of innate myeloid cells in the colon during UCLD induction (74). Meanwhile, the percentage of neutrophils in the HTX-offspring induced or not with UCLD was similar to the Control-offspring induced with UCLD (**Figure 5C**). Thus, this study shows that the consequences of gestational HTX in the male offspring surpass the CNS, supporting that gestational HTX can predispose the male offspring to have accelerated and exacerbated UCLD symptoms. We believe that gestational HTX will also impact the symptomology of UCLD in females as it has been observed after EAE induction (21) and hMPV infection (22). Along these lines, it would be important to perform retrospective studies with human patients suffering from immune-mediated diseases, looking for an association with a possible HTX during pregnancy. These studies could contribute important information about whether gestational HTX in humans can be a risk factor for the offspring’s predisposition to exacerbated detrimental inflammatory responses and immune-mediated diseases.

## Conclusion

In mice, gestational HTX impacted the onset and intensity of UCLD in the male offspring, suggesting that gestational HTX could be a potential risk factor for developing a more intense inflammatory disease like UC in humans. The reduced content of GCLC and MUC-2 mRNA and the high percentage of Th17 lymphocytes in the colon of the HTX-offspring can be part of the mechanisms altered in the HTX-offspring, making them more sensitive to an intense inflammatory disease. We emphasize the importance of elucidating the molecular mechanisms that have been affected in the HTX-offspring, aiming to uncover ways to revert or prevent the negative consequences of inflammatory diseases in this progeny.

## Data availability statement

The raw data supporting the conclusions of this article will be made available by the authors upon request to the corresponding author, without undue reservation. 

## Ethics statement

The animal study was approved by Comité de ética Universidad Andrés Bello. The study was conducted in accordance with the local legislation and institutional requirements.

## Author contributions

JR: Investigation, Writing – original draft. MO: Writing – original draft, Conceptualization, Data curation, Writing – review & editing. RH: Writing – original draft. OA: Writing – original draft. MM: Writing – original draft. EC: Writing – original draft. SG: Data curation, Writing – review & editing. KB: Writing – review & editing. SB: Writing – review & editing. PG: Writing – review & editing. MN: Writing – review & editing. HB: Writing – review & editing. AK: Writing – review & editing. CR: Writing – review & editing, Conceptualization.
