# Supplementary material for: Transient gestational hypothyroxinemia accelerates and enhances ulcerative colitis-like disorder in the male offspring
Source: Front Endocrinol (Lausanne). 2024 Jan 4;14:1269121. doi: 10.3389/fendo.2023.1269121 (PMC10794346; doi:10.3389/fendo.2023.1269121)
Supplement: Supplementary file 1 [file DataSheet_1.docx]

**
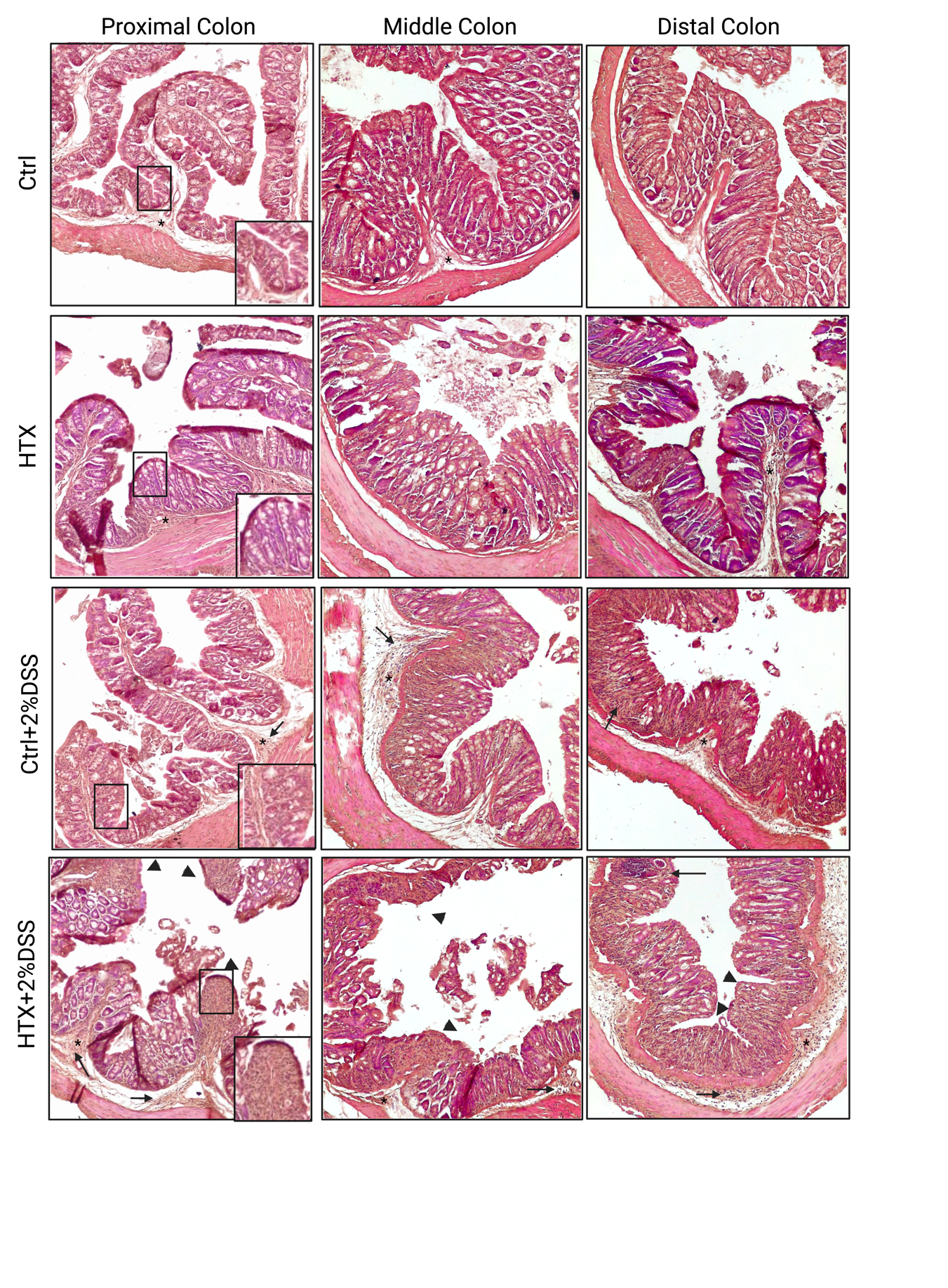
**

**Supplementary Figure 1. Representative images of the colon showing the histopathological analysis of HTX and control offspring suffering or not UCLD**. Optical imaged were taken from the proximal, middle, and distal colon of HTX-offspring and Control-offspring suffering or not UCLD. A digital zoom is shown for proximal colon. The arrows (→) show the inflammation area; the arrowhead (➤) shows mucosa erosion; and the asterisks (*) show inflammatory cell infiltrate in the control and HTX groups without DSS treatment.


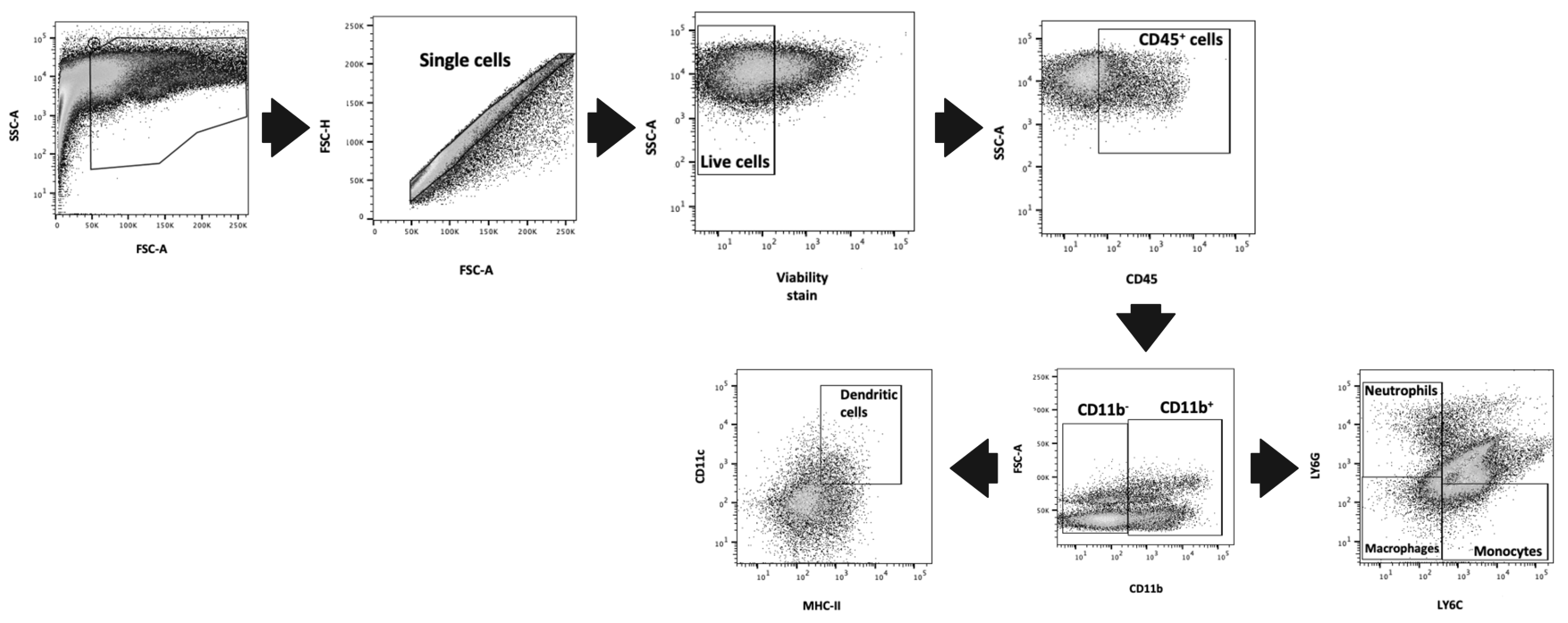


**Supplementary Figure 2. Gating strategy to detect myeloid cell population at the colon. The dot-plot shows the gating strategy to select the cell population**. The first dot-plot identifies the immune cells population, the second dot-plot selects singlet cells; the third dot-plot selects live cells. The fourth dot-plot selects the CD45+ cells; then CD11b+ and CD11b- cells are identified. Over CD11b+ cells, we identified LY6G+ LY6C- (neutrophils), LY6G- LY6C- (macrophages), and LY6G- LY6C+ (monocytes). Over CD11b- cells, we identified CD11c+ MHC-II+ cells (dendritic cells).


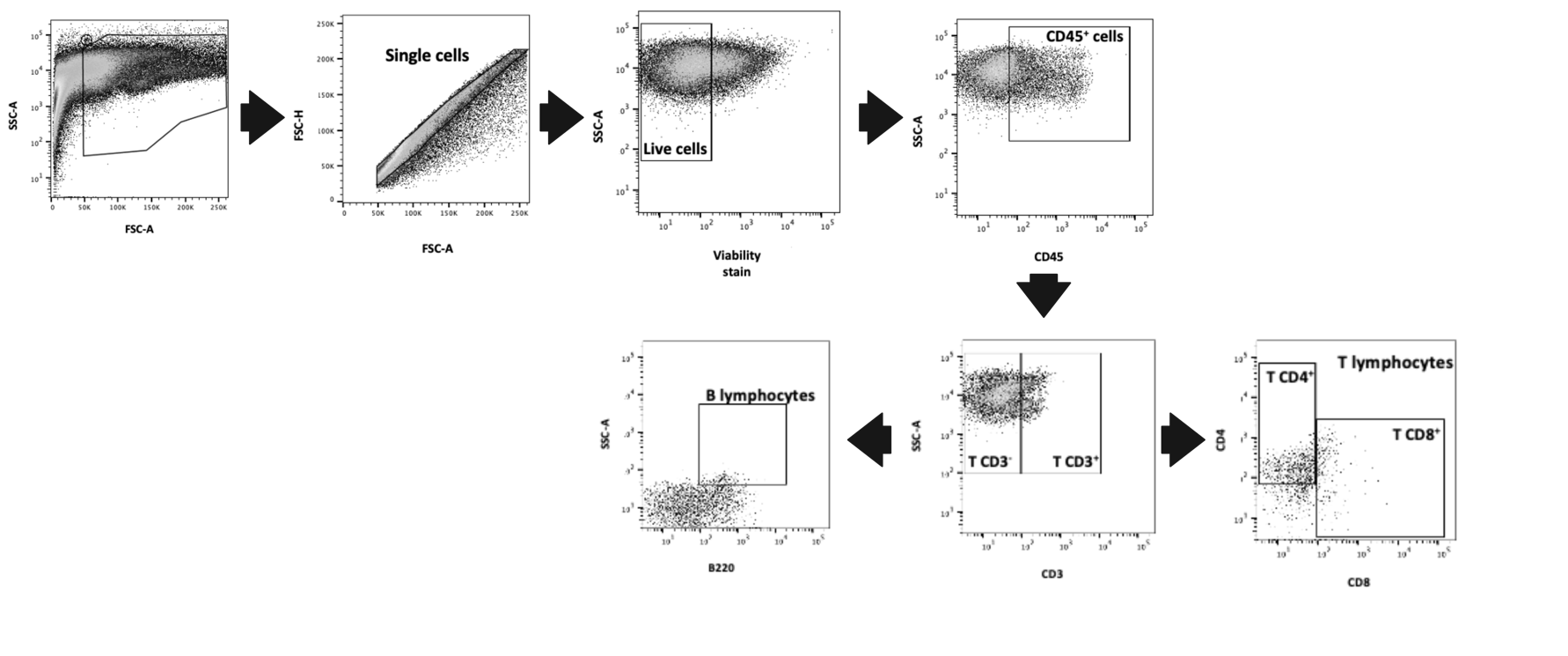


**Supplementary Figure 3. Gating strategy to identify B and T lymphocytes at the colon. The dot-plot shows the gating strategy to identify B and T lymphocytes from the colon samples.** The first dot-plot identifies the immune cells population, the second dot-plot selects singlet cells; the third dot-plot selects live cells. The fourth dot-plot selects the CD45+ cells. Then CD3+ and CD3- lymphocytes are selected from CD45+ cell population. CD4+ or CD8+ T lymphocytes were selected based on CD3+ T lymphocytes. B lymphocytes B220+ were identified based on CD3- lymphocytes.


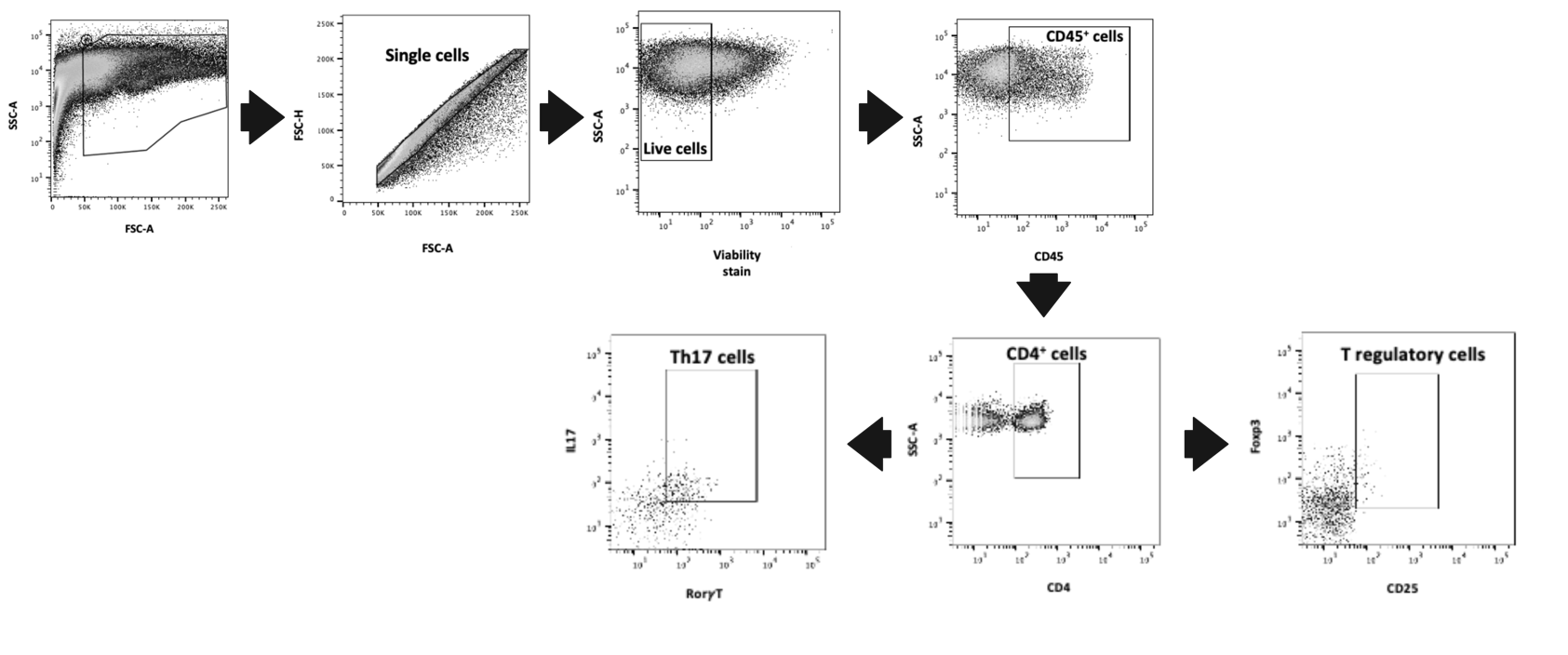


**Supplementary Figure 4. Gating strategy to identify TH17 and Treg lymphocytes at the colon. The dot-plot shows the gating strategy to identify TH17 and Treg lymphocytes from the colon samples**. The first dot-plot identifies the immune cells population, the second dot-plot selects singlet cells; the third dot-plot selects live cells. The fourth dot-plot selects the. Then CD4+ were selected fromCD45+ cells population. From CD4+ T lymphocytes IL17+ and Ror$\gamma$T+ cells were selected to identified Th17 lymphocytes population. From CD4+ T lymphocytes Foxp3+ and CD25+ cells population were identified to select Treg lymphocytes.

**Supplementary Figure 5**: **Correlation matrix analysis**. The figure shows a correlation analysis between DAI and the relative expression of MUC-2, Lipocalin-2, GCLC and the percentage of TH17 cells. A median positive correlation was observed between lipocalin-2 relative expression and DAI. Pearson´s correlation was considered significant when ***p<0.001.

**Supplementary Table I: Clinical score for colitis mice model**

|  | Score of feces | Occult blood |
| --- | --- | --- |
| 0 | Normal and well formed | Negative (No blood detected) |
| 1 | Loose stool | Positive + (Traces) |
| 2 | Watery stool | Positive ++ (moderated) |
| 3 | Watery stools with visible rectal bleeding | Positive +++ (Gross bleeding) |
| 4 | Tenesmus (straining at stool) | - |

**Supplementary Table 2. Histopathological scoring for intestinal tissue**

| Pathological characteristics | Score (in parentheses) |
| --- | --- |
|  |  |
| Distortion of Architecture | Presence (1)  Absence (0) |
| Epithelial Dedifferentiation | Presence (1)  Absence (0) |
| Cryptitis | Presence (1)  Absence (0) |
| Microabscess | Presence (1)  Absence (0) |
| Erosion | Presence (1)  Absence (0) |
| Ulcer | Presence (1)  Absence (0) |
| Granuloma | Presence (1)  Absence (0) |
| Inflammatory infiltrated | Presence (1)  Absence (0) |
| Gravity Infiltrated | Slight (1)  Mild (2)  Severe (3) |
| Localization Infiltrated | Mucosa (0)  Submucosa (1)  Lamina propria (2)  Muscular (3) |

**Supplementary Table 3. qPCR primers**

| Gene | Primers | Forward 5’-3’ | Reverse 3’-5’ | Product (pb) | Tm (ºC) |
| --- | --- | --- | --- | --- | --- |
| *Cat* | CATALASE | CCTCCTCGTTCAGGATGTGGTTTTC | CGTGGGTGACCTCAAAGTATCCAAA | 121 | 60 |
| *Gclc* | GCLC | CCGACCAATGGAGGTGCAGTT | TCTCGTCAACCTTGGACAGCG | 127 | 60 |
| *Gpx1* | GPX | GTCCACCGTGTATGCCTTCT | TCTGCAGATCGTTCATCTCG | 151 | 57 |
| *Hmox1* | HO-1 | CAACATTGAGCTGTTTGAGGAGC | TGTCTCTGCAGGGGCAGTAT | 129 | 57 |
| *Nfe2l2* | NRF2 | TAGATGACCATGAGTCGCTTGC | CGGTATTAAGACACTGTAATTCGGG | 194 | 55 |
| *Nos2* | iNOS | CAGCTGGGCTGTACAAACCTT | CATTGGAAGTGAAGCGTTTCG | 94 | 57 |
| *Nqo1* | NQO1 | AATGACATCACAGGTGAGCTGAA | GCAATGGGAACTGAAATATCACCA | 153 | 55 |
| *Muc2* | MUC-2 | AGAACGATGCCTACACCAAG | CATTGAAGTCCCCGCAGAG | 131 | 55 |
| *Lcn2* | Lipocalin-2 | AAATTGCACAGGTATCCTCAG | CAGAGAAGATGATGTTGTCGT | 218 | 57 |
| *Rps6* | RPS6 | GAAGCGCAAGTCTGTTCGTG | GTCCTGGGCTTCTTACCTTCT | 227 | 57 |
